# Somesthetic, Visual, and Auditory Feedback and Their Interactions Applied to Upper Limb Neurorehabilitation Technology: A Narrative Review to Facilitate Contextualization of Knowledge

**DOI:** 10.3389/fresc.2022.789479

**Published:** 2022-03-01

**Authors:** Camille E. Proulx, Manouchka T. Louis Jean, Johanne Higgins, Dany H. Gagnon, Numa Dancause

**Affiliations:** ^1^School of Rehabilitation, Faculty of Medecine, Université de Montréal, Montreal, QC, Canada; ^2^Center for Interdisciplinary Research in Rehabilitation of Greater Montreal – Site Institut universitaire sur la réadaptation en déficience physique de Montréal, CIUSSS Centre-Sud-de-l'Île-de-Montréal, Montreal, QC, Canada; ^3^Department of Neurosciences, Faculty of Medecine, Université de Montréal, Montreal, QC, Canada; ^4^Centre interdisciplinaire de recherche sur le cerveau et l'apprentissage (CIRCA), Université de Montréal, Montreal, QC, Canada

**Keywords:** augmented feedback, neurorehabilitation, upper limb, virtual reality, robotics, plasticity, stroke, hand

## Abstract

Reduced hand dexterity is a common component of sensorimotor impairments for individuals after stroke. To improve hand function, innovative rehabilitation interventions are constantly developed and tested. In this context, technology-based interventions for hand rehabilitation have been emerging rapidly. This paper offers an overview of basic knowledge on post lesion plasticity and sensorimotor integration processes in the context of augmented feedback and new rehabilitation technologies, in particular virtual reality and soft robotic gloves. We also discuss some factors to consider related to the incorporation of augmented feedback in the development of technology-based interventions in rehabilitation. This includes factors related to feedback delivery parameter design, task complexity and heterogeneity of sensory deficits in individuals affected by a stroke. In spite of the current limitations in our understanding of the mechanisms involved when using new rehabilitation technologies, the multimodal augmented feedback approach appears promising and may provide meaningful ways to optimize recovery after stroke. Moving forward, we argue that comparative studies allowing stratification of the augmented feedback delivery parameters based upon different biomarkers, lesion characteristics or impairments should be advocated (e.g., injured hemisphere, lesion location, lesion volume, sensorimotor impairments). Ultimately, we envision that treatment design should combine augmented feedback of multiple modalities, carefully adapted to the specific condition of the individuals affected by a stroke and that evolves along with recovery. This would better align with the new trend in stroke rehabilitation which challenges the popular idea of the existence of an ultimate good-for-all intervention.

## Introduction

Stroke is defined as an interruption of blood supply in the brain that causes neural damages. It is a common neurological event that often alters the integrity of the sensorimotor system (1) and affects the ability to use the upper limb. Most individuals who sustain a stroke are challenged by a reduction of hand dexterity and hand-related functional capacities (2–5). Hence, recovery of hand function often is a priority in rehabilitation. Successful sensorimotor training relies on task-specific practice in which individuals affected by a stroke engage in meaningful goal-directed tasks with the intention of acquiring new skills (6). Importantly, the tasks trained should be challenging enough to require learning (7), be adaptable in order to calibrate to the person's progress, be intensive (e.g., elevated number of repetitions) and practiced rapidly after a stroke. These tasks should also require attention and focus (i.e., active participation) from the subject (8–10).

The benefits of meaningful task-specific practice can be seen through behavioral changes. For instance, subacute stroke individuals receiving task-specific practice were shown to recover better than the ones treated with a conventional exercise regime (i.e., passive, active-assisted and active movements, stretching, strengthening and coordination exercises) (11). Impressively, significant effects were observed across multiple evaluation tools including the Wolf Motor Function Test (WMFT), the motor portion of the Fugl-Meyer Assessment (FMA) and the hand function domain of the Stroke Impact Scale (SIS). These findings highlight the importance of learning and practicing meaningful task-specific activities to increase rehabilitation efficacy after stroke (12, 13).

Despite rehabilitation, only 5 to 20% of individuals affected by a stroke regain satisfactory hand function while over 60% remain unable to use their paretic hand 6 months after stroke (14). Rehabilitation professionals and scientists are constantly searching for ways to enhance rehabilitation efficacy and improve hand recovery, with a rapid emergence of promising technologies. Individuals affected by stroke can now benefit from several technology-based interventions, among the most prominent ones being virtual reality and upper extremity exoskeletons, including soft robotic gloves (15). These novel approaches are well-aligned with key principles of adaptive neuroplasticity and neurorehabilitation (6, 16, 17). In addition to favoring practice-based learning, they can provide augmented sensory feedback targeting various systems (i.e., somesthetic, visual and auditory) to improve hand function. As such, they offer new possibilities to develop effective rehabilitation catalysts that could improve stroke recovery. The objective of this paper is to review the basic knowledge on post lesion plasticity and sensorimotor integration processes in the context of augmented feedback in virtual reality and soft robotic gloves used for post-stroke rehabilitation of the hand. Also, some factors to consider for incorporation of augmented feedback in the development of technology-based intervention in rehabilitation will be addressed.

## Plasticity in the Motor and Somatosensory Cortex

Brain injuries, such as the ones caused by stroke, trigger multiple anatomical and physiological changes. At the anatomical level, axonal sprouting in periinfarct tissue (18, 19) can favor the reorganization of the motor system and recovery. Following brain injury targeting forelimb motor representations in adult rats, there is a reorganization of the pattern of corticospinal projections (20). Projections from the hindlimb cortex reorganize to reach cervical motoneuronal pools so that it “takes over” the control of the forelimb. These anatomical changes correlate with recovery of the forelimb function. Axonal sprouting can also be observed in remote cortical areas in the ipsilesional hemisphere and even in contralesional brain regions (21, 22). The involvement of these distant, spared areas is affected by the volume of the injury. For example, several studies suggest that the premotor cortex could play a greater role in recovery after larger lesions. Premotor areas are involved in the planification and production of movements. They have direct projections to the spinal cord and are interconnected with M1 (23–26). In monkeys, it was shown that premotor areas can form new corticospinal connections (27) after brain injuries or new cortical connections with the periinfarct tissue and somatosensory areas in the parietal cortex (28). This rewiring could help the premotor cortex to compensate for the functional loss caused by the injury. At a physiological level, the motor cortex is organized in functional topographic maps in which a specific cortical territory evokes movement in different parts of the body (29). During normal development, maps form in a proximal to distal sequence and extensively change, and these phenomena correlate with the acquisition of various skills (30, 31). The borders between body segment representations are defined by reciprocal inhibition between representations (32) and they reorganize in response to experience and training (33). Reorganization of motor maps has been shown in both animal models and humans (9, 34) and is observable in both intact and injured brains (35). However, a study in adult, uninjured monkeys demonstrated that simple repetition of a motor task is not sufficient to produce plasticity of cortical motor maps. The task must be challenging enough so that improvement of performance and learning can occur with movement repetition (7). When a brain injury occurs in M1, it leads to the disorganization of the representational maps in the perilesional cortex, probably due to a disturbance in the reciprocal inhibition between representations (33, 36). Eventually, a spontaneous recovery occurs, and is associated with neurophysiological reorganization. As for anatomical changes, results in animal models suggest that physiological reorganization after smaller lesions may rely more on perilesional plasticity and that recovery after lesions of bigger volume are accompanied by reorganization in other, more distant areas, including the premotor cortex (37–40). This extraordinary capacity of motor maps to reorganize could provide a substrate to exploit in rehabilitative approaches after stroke. For example, rehabilitation based on repetition of movements and learning in monkeys was shown to induce cortical reorganization in the perilesional cortex, likely to support the recovery of hand function (33). Similarly in humans, the addition of a therapy incorporating repetitive practice of a novel task involving only the more affected arm expands ipsilesional motor map area of the extensor digitorum communis muscle (41). It is also worth noting that the effects of rehabilitation on the reorganization of representation maps have been shown to occur early after stroke in animal studies (33). Hence, rapidly starting rehabilitation following a stroke may be crucial to maximize the potential of therapy.

As in the motor cortex, topographic maps of the primary somatosensory (SI) cortex reconfigure after S1 lesions (42). Although perhaps often overlooked clinically, injuries in the somatosensory cortex have been shown to have a profound impact on motor function, in both animal models and humans (43, 44). For instance, in monkeys, removal of the digit representation in S1 resulted in difficulties with tactile discrimination but also clear functional deficits on a motor task that required precise finger dexterity (42). In addition, pharmacological inactivation of S1 impairs finger coordination, the adequate positioning of fingers and the control of grip forces (45). The contribution of the somatosensory cortex to movement control can also be observed in brain stimulation studies in humans. Several studies using non-invasive stimulation over S1 show positive effects on motor learning (44, 46–48). Not surprisingly, plasticity or reorganization in S1 can affect recovery. A study in humans demonstrated that following a hemiparetic stroke, motor recovery was linked to an increased responsiveness of the somatosensory cortex to somatosensory input provided through tactile stimulation (49). Thus, novel technologies aiming to improve functional recovery after stroke should aim to exploit plasticity in both sensory and motor systems.

## Sensory Integration

### Somatomotor Integration

**Figure 1:**
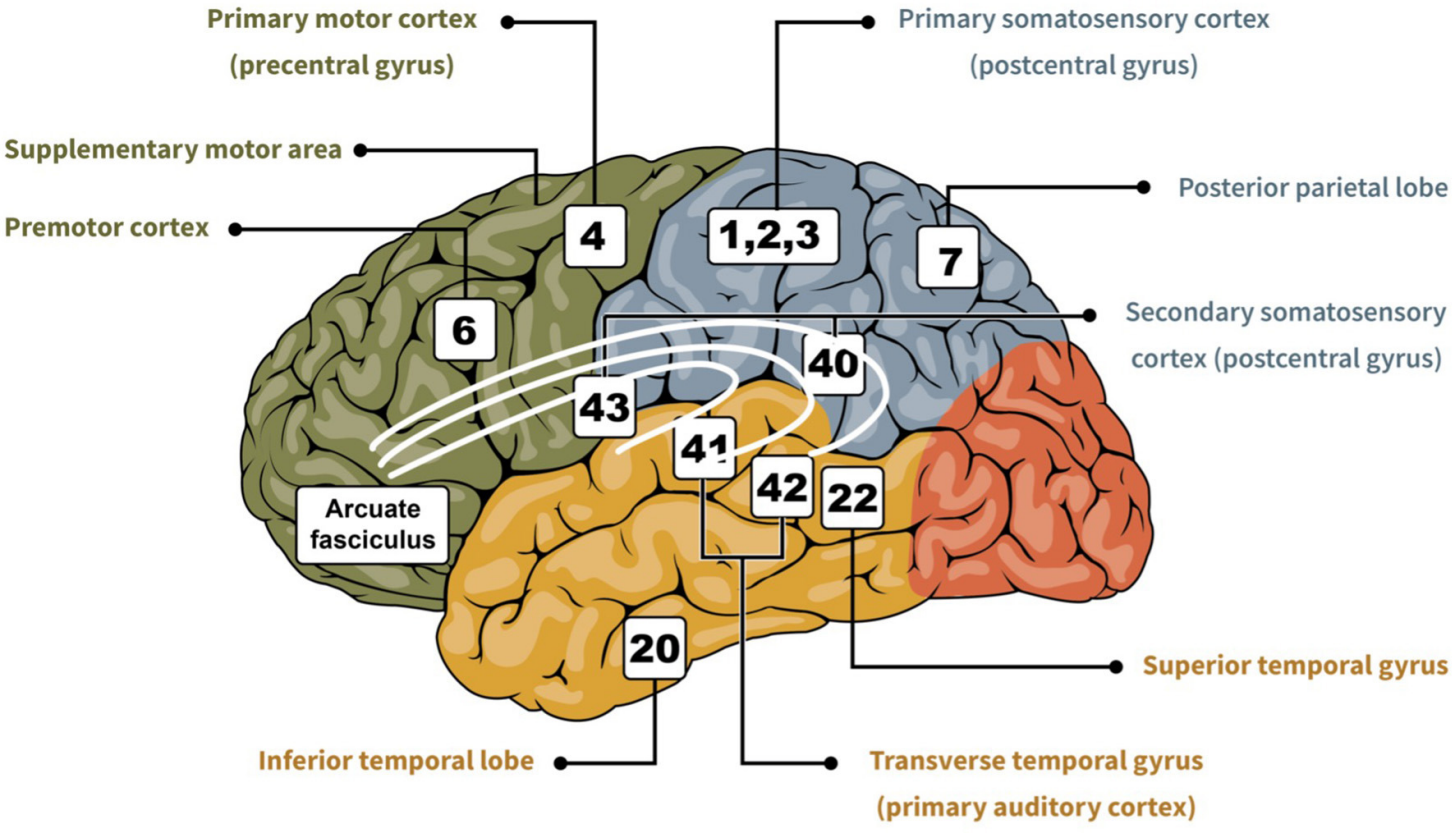
Cartoon showing important regions of the brain related to sensorimotor integration (i.e., somesthetic, visual, and auditory). In green is the frontal lobe, in blue is the parietal lobe, in red is the occipital lobe, and in yellow is the temporal lobe. The numbers indicate the area based on the Brodmann classification (64), and each is associated with their respective designation.

Sensory inputs allow us to interact with our environment by providing feedback to the motor system for successful motor behaviors (50). For the somesthetic system, that feedback originates from cutaneous and proprioceptive receptors, and reaches the parietal cortex (51). Cutaneous inputs are important for the fine-tuning of dexterous movements. They allow individuals to engage in manual tasks of everyday life, ranging from grasping objects to playing musical instruments (52). Proprioceptive inputs are critical for motor planning and motor adaptation of the upper extremity (53). In general, S1 detects and localizes sensory information and the secondary somatosensory area plays a role in focusing attention on somatosensory stimuli (54). Tracer injections in M1 in monkeys have revealed that M1 is extensively interconnected with somatosensory areas (e.g., 3a, 1, 2, and S2) (24, 55). The close relationship that exists between somesthetic inputs and motor performance is likely supported by these direct, reciprocal projections between the somatosensory and motor areas. These projections can be used by the somatosensory cortex to modulate the excitability of the motor cortex (56–58). There are some differences of cortical connectivity between rostral and caudal portions of M1, and they potentially indicate different functional roles subregions of M1 take for the production of movements. Another series of studies in monkeys have shown that the rostral part of M1 receives more proprioceptive inputs (59) and that lesions in this region primarily lead to aiming imprecision (60). In contrast, the caudal part of M1 receives more cutaneous inputs and lesions in this region primarily lead to sensory monitoring deficits. Lesions in the parietal lobe may compromise the integration of somatosensory feedback that can affect movement production (61). For example, a study on 20 individuals who sustained an acute parietal stroke without thalamic involvement, nor visual field deficit, and little to no motor weakness classified three types of deficits according to the location of the lesion in the parietal cortex: (1) lower-anterior parietal stroke resulting in impaired faciobrachiocrural touch, pain, temperature and vibration; (2) superior-posterior parietal stroke resulting in isolated loss of discriminating sensation; and (3) parietal lesions of different topography resulting in sensory loss of all modalities of sensation in a partial distribution. Finally, there is some support for hemispheric specialization in humans. For example, individuals with a lesion to the right parietal lobe experience more difficulty with spatial relationships, suggesting a predominant role of this hemisphere in the representation of the body in the environment (62). Individuals with a lesion in the left parietal lobe experience difficulty executing movements and performing complex spatial tasks, suggesting a predominant role of this hemisphere in the ability to internally represent movement and actions (63). A good understanding of the sensory integration processes and how they have been affected by the stroke is important (Figure 1). It can help determining which therapeutic interventions are most likely to be beneficial according to the type of deficits.

### Visuomotor Integration

Cortical processing of visual inputs is organized in two distinct pathways originating in the primary visual cortex located in the occipital lobe (65). The ventral visual stream projections terminate in the inferior temporal lobe for perceptual identification, while the dorsal visual stream projections terminate in the posterior parietal regions to mediate the required sensorimotor transformations for actions. Through the dorsal stream, visually guided hand reaching and grasping can thereafter be mediated by connections between the parietal regions and the premotor cortex, PMv and PMd (66). From lesion studies, visual functional hemispheric lateralization also appears to exist. Right hemisphere lesions are more associated with visuospatial deficits (67), whereas left hemisphere lesions are more associated with visuomotor adaptation impairments (68). Observing actions can also engage cortical areas responsible for motor execution through the Mirror Neurons System (69, 70). Typically, mirror neurons are activated when both performing an action or when observing that action performed by another individual (71, 72). These neurons were discovered in monkeys with invasive recording methods. However, several neurophysiological and brain-imaging experiments support that this system also exists in humans. For example, activation of regions of the frontal and parietal cortices related to action observation was shown using fMRI (73). Among many potential roles, the Mirror Neurons System is believed to be crucial to use visual information, in particular action-related observations, to reinforce or form brain activation patterns that can generate these actions. This strong link between action observation and generation can be exploited in post-stroke rehabilitation of the hand.

### Audiomotor Integration

Auditory inputs reach the external ear, then hair cells in the cochlea for acoustic frequencies analysis (74). Through hair cells, auditory nerve fibers project to the cochlear nuclei in a tonotopic organization, then to superior olivary nuclei and converge midbrain in the inferior colliculus. Projections from the inferior colliculus reach the thalamocortical system for auditory cortical processing. Some studies suggest similar sensory processing streams as in the visual system (65), where distinct pathways are specialized in object localization and object identification, despite some overlap (75–77). For example, an fMRI study in humans suggests that the anterolateral transverse temporal gyrus, anterior superior temporal gyrus and posterior planum polare process auditory object identity and that the planum temporale and posterior superior temporal gyrus process auditory object location (78). Knowledge of this segregation can be particularly useful to better predict the sensory deficits that individuals may have following brain injuries in these respective regions. As in the visual system, auditory neglect, would be associated with right brain lesions to the parietal lobe or the thalamus (79). Moreover, the contribution of the Mirror Neurons System could also be involved in the recognition of auditory inputs (71, 80, 81). For example, neuronal recordings from PMv in monkeys have revealed some mirror neurons selective to both auditory and visual cues, but also others that are selective to only auditory or only visual cues during the same hand-related action (80). This suggests that even in the absence of visual input, mirror neurons can discharge solely by relying on auditory inputs related to a specific action. A human study using transcranial magnetic stimulation (TMS) supports the influence of action-related sounds on M1 excitability. Subjects were assigned to three groups that had to listen to either hand action sounds (i.e., typing or tearing paper), leg action sounds (i.e., walking), or controlled noise unrelated to an action (i.e., thunder). When listening to these sounds, stimulations were delivered to M1 while recording outputs to the first dorsal interosseous. The results showed that sounds associated with hand-related actions produced greater corticospinal motor outputs than leg-related or random sounds (82). This study underlines the potential contribution of movement related sounds to movement production, likely occurring through the Mirror Neurons System (83). Thus, the auditory system has an important role to play in interpreting action and object-related sound that should be considered in neurorehabilitation and rehabilitation technologies.

### Multimodal Sensory Integration and Stroke

**Figure 2:**
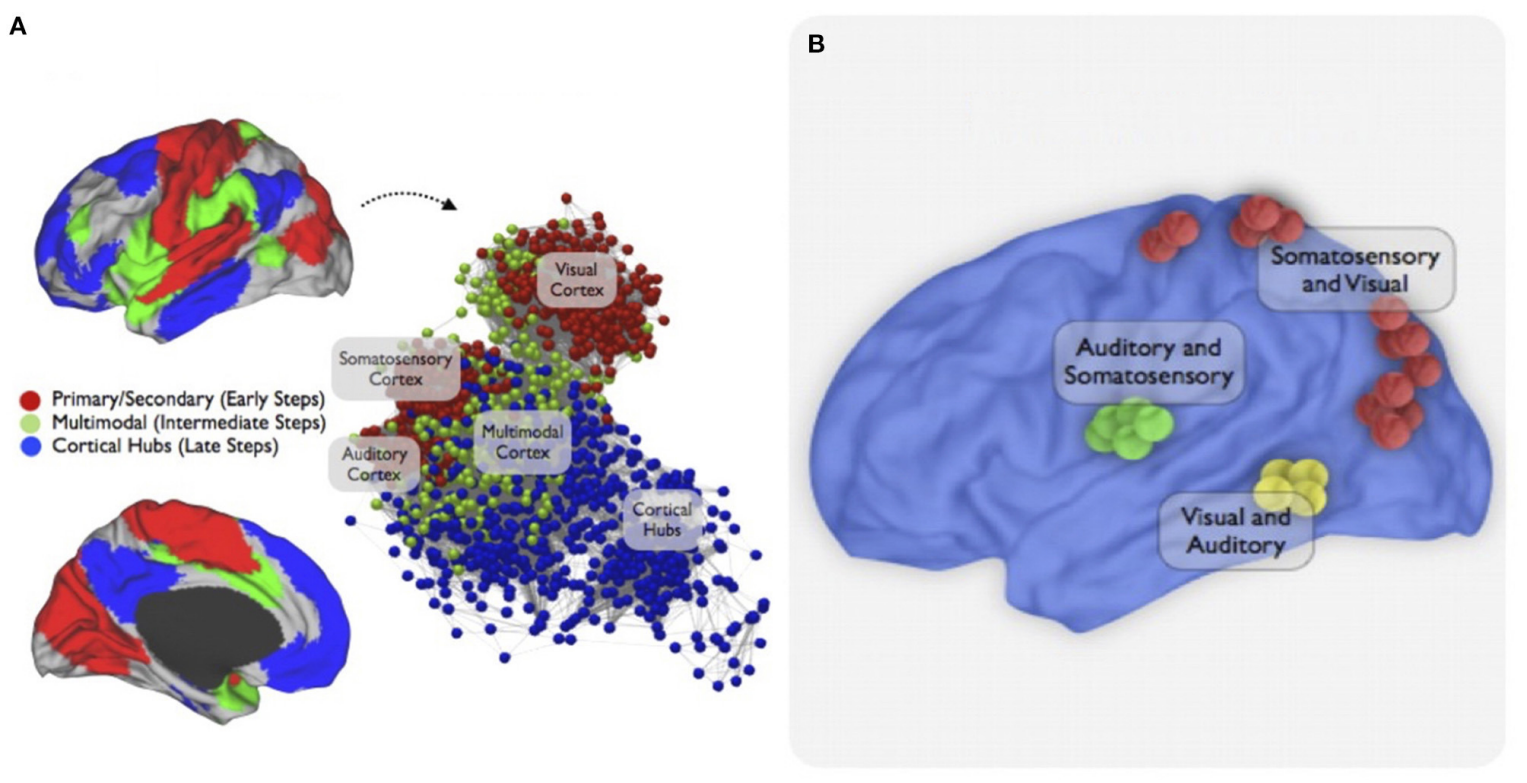
Multimodal integration network. This figure is from a study that used resting-state functional connectivity MRI and stepwise functional connectivity (SFC) analysis to investigate sensory integration networks in the human brain. SFC patterns of primary cortices were first explored to target main convergence regions of multimodal integration. Then, a combined approach to highlight the topological convergence of the stepwise connectivity patterns in the three major sensory modalities was used. **(A)** The combined SFC map of connectivity patterns of brain regions of all modalities using a seed-based approach is shown. The sensory integration begins in the unimodal-related systems (early stages/red nodes) then converges in the multimodal integration network (intermediate stages / green nodes) before joining the cortical hubs (late stages / blue interface). An energy layout algorithm that considers the difference between geometric and pairwise shortest-path distances of nodes resulted in the network graph displays. **(B)** Finally, an interconnector network analysis explored specific functional connectivity profiles between pairs of sensory cortices showing bimodal integration regions between somatosensory, visual, and auditory cortices in the human brain. Figure from Sepulcre et al. (86).

Projections from the parietal lobe to the motor cortex encode and integrate sensory information from somesthetic, visual and auditory stimuli (84, 85). Further divisions of M1 based on connectivity have been proposed in capuchin monkeys, leading to the hypothesis that M1's territory is formed of different modules, each primarily interconnected with a distant cortical area of the sensorimotor network (24, 25). This modular organization could sustain parallel processing of multiple input sources and subsequent integration of this information to increase the behavioral repertoire of the hand. In humans, functional connectivity analyses show that visual, somatosensory and auditory information converge in multisensory integration centers, creating a frontoparietal network (Figure 2) (86, 87). However, the multimodal integration hubs are not the destination, but rather serve as transition zones toward regions of the brain involved in cognition. In multimodal zones, the impact of inputs from one modality can be modulated by input from another modality. In particular, if two congruent stimuli are presented simultaneously or with short latencies, the evoked response is greater, and this is likely to favor detection and appropriate response to the event (51). Multiple examples of integration of signals across sensory modalities exist. For example, when participants are asked to indicate the presence or absence of a brief, low-intensity sound presented alone or in combination with simultaneous light, there is an improvement of detection of the auditory stimulus in the presence of the light (88). Moreover, brain injuries that affect the parietal lobe can impair the integration of more than one sensory signal (61, 68, 79), highlighting its role in multisensory integration. Because various sensory inputs can reinforce each other, the integration of different sensory modalities in rehabilitation interventions could be more powerful than using a single modality of feedback. Because they allow to amplify several modalities and multisensory integration, virtual reality and soft robotic gloves should be promising neurorehabilitation interventions.

## Stroke Rehabilitation and Sensorimotor Augmented Feedback

The close relationship that exists between sensory inputs and motor behavior makes sensory feedback a potential asset for rehabilitation of the upper limb after stroke. Augmented sensory feedback can be integrated into hand rehabilitation in several forms which will be discussed in this section. Indeed, somesthetic feedback can be integrated through haptic feedback and somatosensory stimulation. Visual feedback is incorporated in action observation therapy, in mental practice therapy, and in mirror therapy. Finally, auditory feedback is often integrated, but not exclusively, into two forms: rhythmic auditory cueing and kinematic auditory feedback. All of these feedback modalities can be used in technology-based interventions, such as virtual reality and soft robotic gloves to enhance recovery of sensorimotor function after stroke.

Virtual reality is a computer-based technology in which users are immersed in a multisensory simulated environment to imitate real-world interactions. Virtual reality systems vary based on the degree of immersion and the type of sensory feedback used. A fully immersive system changes visual perspective with head movements, while a semi-immersive system offers a fixed visual perspective in three dimensions. Finally a non-immersive virtual reality system offers a fixed visual perspective in two dimensions (89). Although they provide better and more realistic feedback, fully immersive systems are more likely to cause health and safety risks, such as dizziness and sickness (90, 91). When simulating real objects or interacting with real objects, virtual reality provides the user with augmented feedback of various modalities, particularly visual and auditory. Somesthetic feedback can be precisely controlled when adding a soft robotic glove. These gloves may be particularly useful in rehabilitation to enable people with somatosensory-motor deficits. There are several models of gloves that vary, among other things, according to the type of actuator (i.e., motor, elastic, pneumatic), the power transmission, the intention detection method and the capacity to generate a movement (e.g., passive, active-assisted) (92, 93).

### Somesthetic Augmented Feedback in Rehabilitation

Somesthetic feedback can come in two main forms, somatosensory stimulation, and haptic feedback. Epidural spinal cord stimulation and intracortical microstimulation of the somatosensory cortex have been used to provide artificial somatosensory feedback (94, 95). However, the most common approach to deliver somatosensory stimulation is with peripheral nerve stimulation (PNS). PNS consists of low-intensity electrical pulses that are typically delivered to the median, the ulnar or the radial nerve and can also be used for functional electrical stimulation in the context of hand rehabilitation (96). In healthy individuals, PNS has been shown to activate S1 and increase M1's excitability (97). After stroke, PNS could potentially be used to induce cortical reorganization of the motor and somatosensory cortices and improve functional recovery. This hypothesis has been supported by studies after both acute and chronic stroke, thereby making PNS a promising therapeutic tool for rehabilitation of hand deficits (98–100). A randomized study of chronic stroke individuals has shown significant hand function improvements after PNS compared to controls (101). Following ten consecutive daily sessions of 2 h of PNS to the radial and median nerves paired with 4 h of intensive task-oriented training, the experimental group showed significantly greater scores on the FMA, the WMFT and the Action Research Arm Test (ARAT). Moreover, only the experimental group showed a significant carry-over effect at 1-month follow-up, in both the FMA and the ARAT.

Haptic feedback refers to the provision of augmented tactile and force stimulation through a physical device (102), hence its relevance for soft robotic exoskeleton training and virtual reality (103). Haptic feedback is an integrative aspect of soft robotic exoskeletons via actuators or sensors which offer a sensation of movement to individuals who have little to no mobility in their affected hand (104).

### Visual Augmented Feedback in Rehabilitation

After stroke, visual information and visuomotor integration can be exploited to improve functional recovery. This section will cover three common interventions relying on visual feedback in stroke rehabilitation: action observation therapy, mental practice, and mirror therapy. Action observation therapy generally consists of two phases, action observation sessions and execution sessions. In individuals with stroke, action observation therapy activates many areas of the Mirror Neurons System as well as other areas involved in motor execution (69). The activation pattern is generally bilateral, although some studies have shown that the effects can also be limited to the ipsilesional hemisphere (105, 106). The therapy has been reported to induce significant and meaningful positive effects after stroke. For example, subacute stroke individuals (<3 months) that received 8 weeks of action observation therapy improved significantly more on the FMA and the Barthel Index (BI) in comparison to the group that received conventional rehabilitation training (70). In the experimental group, the intervention showed positive carry-over effects in all outcomes at 2 month follow-up, with even higher scores than at the end of training. At follow-up, fMRI also showed that the action observation therapy group had a significant increased activation in the precentral gyrus, the parietal lobe and supplementary motor area. This suggests that action observation therapy activated the Mirror Neurons System and led to cortical plasticity in brain regions related to upper limb motor function.

The second approach is mental practice therapy, which consists of repetitive motor imagery in a therapeutic setting. Neuroimaging studies have shown that motor imagery (i.e., mental representation of an action without producing any movements) activates similar brain areas as the production of movement. More specifically when subjects are asked to think of a motor task such as a finger-to-thumb opposition, without producing any movement, this results in increased hemodynamic activity in M1, the premotor cortex and the supplementary motor area (107–109). Mental practice therapy can be distinguished into two types depending on which perspective the person imagines the action: visual imagery if external (i.e., third person) and kinesthetic imagery if internal (i.e., first person). Visual imagery appears to be better for task form, and kinesthetic imagery better for task speed and bimanual coordination (110, 111). Although mental practice therapy has been shown to be effective in enhancing motor performance in healthy populations, these effects are milder than the ones obtained with real physical practice (112). In fact, a systematic review concluded that mental practice therapy alone has no significant effect over conventional treatment after stroke. However, when combined with conventional treatment (113) or action observation therapy (114), it can improve upper limb recovery in comparison to conventional therapy alone.

The third common therapy relying on visual feedback that has received some support in the literature is “mirror therapy.” Mirror therapy is described as a visual illusion created by a mirror that shows the movement of an individual's paretic hand moving normally while moving the non-paretic limb. Although the precise mechanisms through which mirror therapy can favor recovery after stroke are still not well-understood, it is reasonable to propose they are related to the Mirror Neurons System as well (115). In fMRI studies, it was shown that prolonged use of mirror therapy (i.e., over a 6-to-8-week period) can shift the brain activation pattern. When comparing to pretreatment baseline, individuals affected by a stroke had a shift of activity from the contralesional M1 to the ipsilesional hemisphere during movement of the less affected hand, suggesting that the therapy induced neural reorganization (116, 117). This increase of activation in the ipsilesional hemisphere during movements of the non-paretic hand correlates with better motor performance and recovery of hand function (118, 119). Mirror therapy can also be done through virtual reality (120). To date, virtual mirror reality interventions in rehabilitation allow individuals to see a projection of their paretic moving hand, based on the movement of their healthy hand, in a virtual environment. For subacute and chronic stroke individuals, the effects of mirror therapy, including mirror therapy with virtual reality interfaces, confirms significant beneficial effects on motor function and activities of daily living (120). To this day, few studies have compared “classical” and virtual mirror therapy. However, one study in healthy and stroke subjects measured corticospinal excitability using TMS over the flexor carpi radialis cortical representation during repetitive wrist flexion-extension exercise and compared results in both conditions; “classic” and virtual mirror therapy (121). The virtual mirror therapy induced a greater increase of cortical excitability in comparison to the “classic” mirror therapy both in healthy and stroke subjects. Nevertheless, the literature on the additional benefits of virtual mirror therapy compared to standard mirror therapy is still limited. Perhaps the best use of virtual reality in the context of mirror therapy would be with the use of a soft robotic glove (122). In this case, the visual feedback created by the virtual reality interface could be accompanied by assisted movements of the impaired hand by the glove. Combined use of virtual reality and a soft robotic glove would go beyond what mirror therapy can offer.

### Auditory Augmented Feedback in Rehabilitation

Auditory feedback can bring several benefits to rehabilitation. It can increase engagement and motivation, improve memorization, offer guidance during motor tasks (e.g., errors, progress, success) and reinforce realism in virtual reality (123, 124). Auditory feedback interventions typically fall into two broad categories: rhythmic auditory cueing and kinematic auditory feedback, also known as sonification (83). The goal of rhythmic auditory cueing is to synchronize motor execution with a rhythmic sound (e.g., music, metronome). Therefore, the emphasis is on the result/success of the movement or task, known as knowledge of result. From a functional point of view, its therapeutic effects are mostly aimed at increasing the velocity of movement execution (83, 125). As for the kinematic auditory feedback, the purpose is to inform and optimize the movement trajectories, known as knowledge of performance. From a functional point of view, its therapeutic effects target the accuracy of the movement execution (125). Thaut and collaborators did extensive research on rhythmic auditory cueing in rehabilitation after neurological disease, mostly for gait training. They demonstrated its beneficial effects on gait velocity, stride length, and step cadence (126–129). Research on rhythmic auditory cueing for upper limb motor recovery after stroke shortly followed. For example, training with rhythmic cueing (i.e., metronome) during a reaching task improves spatiotemporal control of sequential reaching movement of the impaired arm (130). A recent meta-analysis on auditory feedback for upper limb recovery after stroke revealed that adding auditory training to therapy has beneficial effects on the FMA, SIS, elbow range of motion and WMFT (83). Although beneficial effects were present with both rhythmic auditory cueing and kinematic auditory feedback, kinematic auditory feedback had greater beneficial effects on the FMA. This suggests that auditory feedback, especially focusing on movement trajectories, can be incorporated into all kinds of therapies to increase their beneficial effects on functional recovery. Auditory feedback to monitor a motor task can easily be integrated with virtual reality and soft robotic gloves for stroke rehabilitation training (123, 124, 131). Moreover, virtual reality and soft robotic gloves often integrate other auditory inputs, known as auditory displays, to better interact with the technology. In contrast to auditory feedback, the integration of auditory displays into technologies has no therapeutic aim. They are rather a tool used to facilitate the communication between the user and the device in order to increase usability (124, 132). Three different types of auditory display can be integrated with virtual reality and soft robotic gloves to facilitate user-technology interaction: speech, auditory icons and earcons (132). Speech is a verbal auditory display derived from audio recording or synthetic voices. While this type of auditory display can be very useful for encoding multiple and complex information, it often requires greater cognitive workload for proper interpretation. It also relies on a good knowledge of the language of use, which may limit its usability for some users. Auditory icons refer to the notion of action-related auditory feedback. These are sounds associated with events or actions that produce sounds that we frequently hear in everyday life activities. These sounds will often be easily and intuitively recognizable, although this will depend on the user's exposure to these various daily events and actions. Finally, for more abstract and less intuitive uses, auditory icons are not optimal and earcons are preferred. Earcons are abstract sounds that the user must learn to associate with the desired interpretation. Despite their different aims, it is important to consider both auditory feedback and auditory displays, and their potential interaction in the development of technology-based interventions to avoid overwhelming the user with contradictory or counterintuitive information.

## Unimodal VS. Multimodal Augmented Feedback in Therapy

Based on the previous literature, it can be concluded that unimodal augmented feedback has great potential to have positive impacts on motor function in stroke subjects. However, it has been highlighted that most rehabilitation technologies, such as virtual reality and soft robotic gloves, favor a multimodal approach in terms of augmented feedback. Virtual reality provides an interactive environment for motor training tasks through augmented visual and auditory feedback, while soft robotic gloves mostly, but not exclusively, provides haptic feedback with realistic tactile sensations to improve dexterity and fine motor skills. This combination is also appealing to enhance the experience of individuals who sustained a stroke by providing a more realistic environment, which further increases their level of presence and motivation (133). While designing approaches that simultaneously enhance various forms of sensory feedback is relatively easily achievable with current technology, one may wonder if a multimodal approach should always be privileged over a unimodal approach. In some cases, one form of feedback is clearly more valuable than others. This was shown in a study that compared the value of visual and proprioceptive feedback to guide motor adaptation to perturbations in a reaching task (134). Healthy subjects had to perform a reaching task in the horizontal plane while holding with their dominant arm the handle of a two-joint robot. The robot pushed the hand away from its intended target during trials. Three conditions were compared: concurrent visual and proprioceptive feedback (i.e., cursor reporting the hand location during movement), proprioceptive feedback only (i.e., immobile cursor during movement), and “false visual feedback” (i.e., cursor reporting improperly the hand's location in space during movement). In this latter condition, subject simultaneously received conflictual proprioceptive (e.g., trajectory errors) and visual (e.g., trajectory is correct) feedback. The results demonstrated similar beneficial performance in hand-path errors between concurrent visual and proprioceptive feedback and proprioceptive-only feedback. As for “false visual feedback” results showed a deterioration in hand-path errors. Together, the results suggest no added value of a multimodal approach (i.e., visual and proprioceptive) for guiding reaching tasks and that proprioception alone is sufficient to eliminate directional errors and ensure a smooth hand trajectory. Interestingly, this conclusion was supported by another experiment that compared haptic feedback, visual feedback and visuo-haptic feedback (135). Healthy individuals had to perform a reach-to-grasp task with their right hand toward an object that unexpectedly changed size. In the haptic condition, by holding the object with their left hand, participants could sense the size change. In the visual condition, the change in size was detected by visual information only. The visuo-haptic condition combined both inputs. The results demonstrated that grip aperture correction responses to object size was faster in the haptic-only condition than in visual-only condition. There was no difference between the haptic-only and visuo-haptic conditions. This supports that performance cannot always be predicted based on a simple summation of effects obtained with unimodal feedback sources. In reach-to-grasp tasks, haptic feedback may be more important to prioritize. Nevertheless, there is evidence supporting that the combination of multiple modalities can generate better results. A meta-analysis of 43 studies examined the effects of multimodal feedback on healthy users' performance in motor tasks. They compared visual-auditory and visual-tactile feedback to visual feedback alone (136). Results showed that adding auditory or tactile feedback to visual feedback reduced reaction times and improved accuracy. However the combination of modalities was not effective in reducing error rates. This suggests that visual feedback has more influence on error rates than auditory or tactile feedback, at least in control subjects. After stroke, a study compared the effect of unimodal vs. multimodal feedback (i.e., auditory and visual feedback vs. only visual feedback) and found that the addition of auditory feedback to visual feedback improved trajectory quality (e.g., linearity of the path) on reaching tasks (137). These results suggest that each modality of sensory feedback acts on different parameters of movement production. It therefore becomes interesting to favor the use of multimodal feedback.

When feedback from multiple modalities is congruent in time and space, the efficacy of the combined feedback seems to be greater than unimodal feedback, which is called intersensory facilitation. In fact, the combination effects can be greater than a simple linear summation, known as response amplification (138, 139). In healthy individuals, fMRI revealed a region of the superior temporal sulcus that exhibits a significant supra-additive response to congruent audiovisual feedback. When the audiovisual feedback is incongruous, the response is sub-additive in comparison to unimodal feedback (140). While a multimodal approach can be more effective than a unimodal approach, could it also be detrimental? If multimodal feedback is not congruent, there is potential for response competition. For example, in a rhythmic index finger flexion-extension task (e.g., metronome), healthy individuals were required to touch a physical plastic stop. The stop location was either coincident with or counterphase with the auditory metronome. When the tactile contact with the stop coincided with the metronome, coordination was stabilized as opposed to counterphase feedback (141). This synchrony-dependent mechanism of multimodal feedback integration is useful when the sensory feedbacks from the different modalities occur intermittently, for the detection and identification of a given event. But what occurs when feedback comes in continuously? An experiment in rodents and humans explored the mechanisms of sensory integration when multiple auditory and visual events occur continuously (142). During an audiovisual rate discrimination decision task, subjects had to report their perceived event rates (e.g., high or low) when presented with a series of auditory (i.e., brief sound) or/and visual (i.e., flash of light) stimuli. When presented together, auditory and visual stimuli were either presented synchronously or asynchronously (i.e., independently). The results showed that subjects exhibited significantly better event rates on multimodal stimulus trials than unimodal stimulus trials whether the stimuli were synchronous or independent. This suggests that when feedback comes in continuously, despite the lack of time synchrony, a multimodal approach would be more effective. Altogether, from this literature one could conclude that the risks of competition are relatively limited when using sensory feedbacks from different modalities. This is true when using intermittent feedback with stimuli that are congruent in time and space. In the case of continuous feedback or even asynchronous.

Natural interactions between individuals and the environment predominantly involve multimodal sensory integration processes. It is not surprising that most treatment interventions, involving new technologies or not, rely on multimodal approaches. However, integrating feedback in rehabilitation, whether unimodal or multimodal, must be carefully considered to optimize its impact. In addition to the congruence issues discussed above, the use of multiple feedback in treatment design for stroke individuals should consider the feedback delivery parameters, task complexity and heterogeneity of sensory deficits. These three factors were favored because they are an integral part of the clinical decision-making process of rehabilitation professionals when choosing a therapeutic approach (143). Each of these factors, and their resulting challenges, will be discussed in more detail to inform rehabilitation professionals and researchers who consider integrating, even combining, different augmented feedbacks into their technology-based interventions in rehabilitation.

## Multimodal Feedback Delivery Parameter Selection and Task Complexity

**Figure 3:**
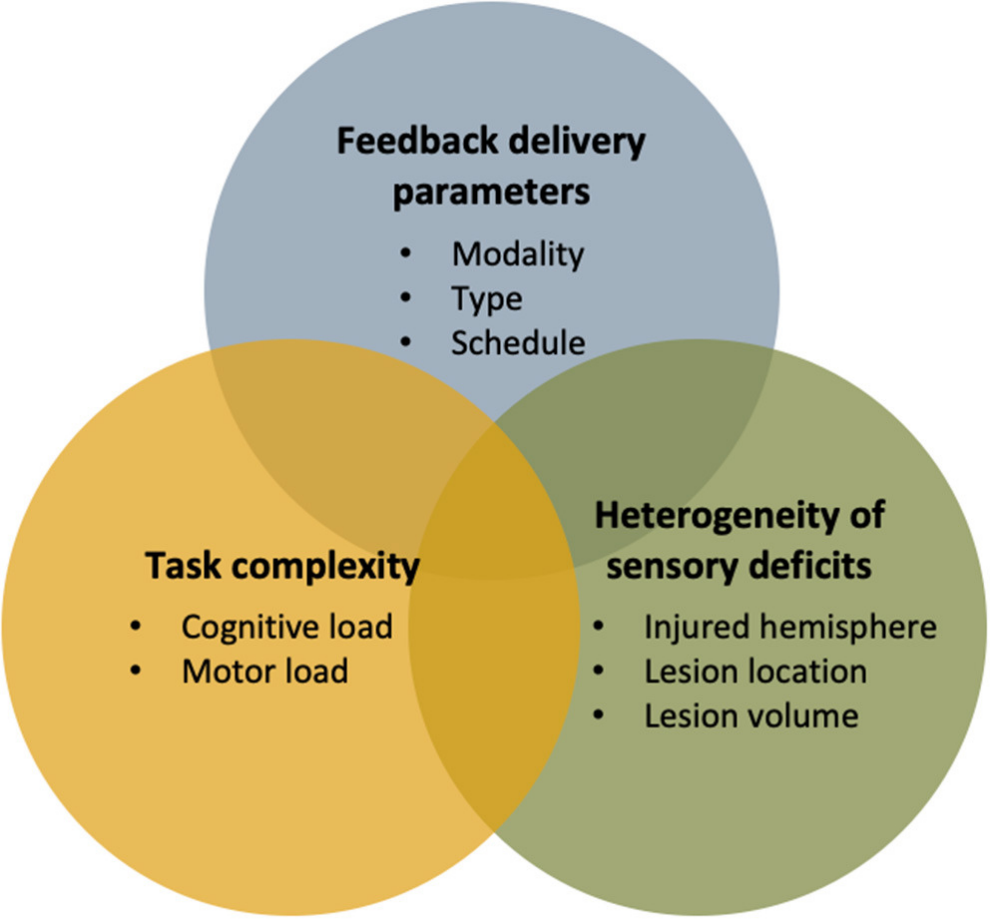
Summary of important factors to consider when designing a multimodal feedback approach in technology-based interventions for sensorimotor rehabilitation.

The design of a treatment incorporating multimodal feedback must consider different delivery parameters, the modality (i.e., somesthetic, visual, auditory), the type (i.e., knowledge of performance/knowledge of result) and the schedule (i.e., continuous/reduced/faded) (Figure 3). To illustrate these parameters, here is an example of auditory feedback that could be incorporated into the design of a simple index finger flexion-extension task. Each time (continuous) the person successfully reached the index finger flexion and extension targets, an auditory cue such as a beep sound is triggered (knowledge of result). As briefly discussed in the *Auditory Feedback* section, it is possible to focus on knowledge of performance and/or knowledge of the result. Knowledge of performance feedback is used throughout the execution of a task (concurrent), whereas knowledge of result feedback is mostly given after completion of the task to inform about the success of the task (terminal). This possibility is not restricted to auditory feedback, it can also apply to visual and somesthetic feedback. Both types of feedback delivery have beneficial effects on motor function but the effects on activity levels are inconclusive (144). A systematic review on the effects of the type of feedback delivery on motor performance after stroke showed that knowledge of performance feedback may lead to greater improvements in arm motor performance and quality of movement with a carry-over effect of at least a month post intervention compared to knowledge of result feedback (145). For knowledge of result feedback, although the effects are lesser, the approach still demonstrates immediate effects on motor performance during the intervention and shows effects on the quality of movement later. Simultaneous use of both types of feedback during the same task can also be performed. Also, a comparative study of knowledge of result feedback after good vs. bad trials on a golf putting task demonstrated that when provided after good performance trials (positive feedback) subjects showed higher levels of self-efficacy and intrinsic motivation, and more accurate performance than after bad trials (negative feedback) (146). This suggests that positive feedback is more beneficial than negative feedback.

The feedback delivery schedule can be continuous, reduced or faded. Continuous feedback is given systematically (100%), reduced feedback is given at a pre-set interval (e.g., every 5 trials), and faded feedback decreases over time (144, 147). A study in healthy adults compared continuous, reduced and faded knowledge of result feedback in an isometric shoulder flexion exercise practiced 20 times a day for 4 days (148). It demonstrated significantly higher error reduction from pre to post intervention in the reduced and faded feedback cohorts. No significant difference was found in the continuous feedback cohorts. These studies suggest that by maintaining the presence of feedback over a long period of time, a dependency can result from its use. This dependence could lead to a “plateau” in rehabilitation by removing the challenging component of motor learning. However, also based on the principles of motor learning, complex tasks increase cognitive and motor load, requiring more assistance in comparison to simple motor tasks (149, 150). The feedback delivery schedule should thus be faded as individuals affected by stroke improve, keeping the task challenging and engaging enough to promote motor learning and recovery. Feedback delivery parameters must be chosen according to the task complexity and the users' abilities (e.g., cognitive and motor). Even for feedback delivery modality, task complexity is another key factor to consider and that should be adapted to the individual affected by stroke. Visuo-auditory feedback is more effective when a task is performed under normal cognitive workload conditions (low complexity), while visuo-tactile feedback is more effective when tasks are performed under high cognitive workload conditions (high complexity; e.g., multiple tasks simultaneously performed in time-constrained scenarios) (136). The choice to favor auditory rather than tactile feedback (i.e., the modality) should depend on task complexity, or the cognitive workload of the task for the individuals affected by a stroke. Further studies are needed to refine the optimal combination between each of the augmented feedback delivery parameters to optimize motor learning after stroke, while considering the impairments of the users and task complexity.

## Heterogeneity of Sensory Deficits

Following brain injury, sensory deficits may vary depending on many factors such as the injured hemisphere, lesion volume and location. A good knowledge of the deficits of an individual affected by a stroke is key to guide the choice of intervention. However, adequately assessing deficits may be challenging. For example, touch and proprioception deficits are often missed due to a lack of consensus in clinical assessment methods, and can lead to errors in diagnosis (151) and inappropriate care. Vision and proprioception are predominant in the perception of spatial information, while hearing is useful in the perception of temporal information related to the periodicity, regularity, and speed of motion. As for augmented somesthetic feedback (e.g., tactile, haptic), it offers both spatial and temporal information, and above all, motor information by directly influencing the orientation or the force of the movement (e.g., force feedback) and reducing trajectory errors (123, 136, 152, 153). Virtual reality and/or soft robotic gloves might be more beneficial for specific lesion types or deficits. In addition to being promising rehabilitation intervention approaches, these tools might favor more precise quantification of sensory deficits. For example, a virtual reality augmented robotic arm can reliably assess upper-limb sensorimotor function after stroke using a visually guided reaching task with greater sensitivity than a standard clinical assessment scale (i.e., Chedoke-McMaster Stroke Assessment Scale) (154). These data will be important to establish a better understanding of the relationship between the effectiveness of sensory-augmented intervention (either unimodal or multimodal) and the type and severity of sensorimotor impairments. A better understanding of the individual and combined effects of each modality would help design interventions best suiting the needs of the patient. Also, comparative studies allowing stratification of the augmented feedback delivery parameters based upon different biomarkers, lesion characteristics or impairments should be advocated. This would better align with the new trend in stroke rehabilitation which challenges the popular idea of the existence of an ultimate good-for-all intervention.

## Conclusion

The existence of an important link between the motor and sensory systems, as well as associative areas of the brain, in the integration sensory feedback is established. However, after a stroke, an alteration of the capability to use and integrate sensory information to produce movements can occur due to the lesion or due to altered connections between brain regions. Therefore, the addition of increased feedback in rehabilitation may prove beneficial in optimizing neurophysiological changes and increasing motor performance. This paper has reviewed current knowledge of the neural mechanisms involved in the interpretation process of the different types of unimodal augmented feedback for upper limb stroke rehabilitation and their integration in a multimodal approach using virtual reality and soft robotic gloves. These technologies can integrate feedback from different sensory modalities and can even combine them together. This multimodal augmented feedback approach seems more promising for rehabilitation, likely for most post-stroke individuals. However, our understanding of multisensory integration mechanisms enabled by these rehabilitation technologies remains limited. Current knowledge does highlight that integrating augmented feedback in rehabilitation requires careful consideration of various factors such as feedback delivery parameters, task complexity and heterogeneity of sensory deficits to maximize the short- and long-term benefits of rehabilitation technologies and technology-based intervention. The methodological design of future research should pay particular attention to these factors.

## Author Contributions

CP conceptualized the research goals and aims. CP and ML conducted the investigation and wrote the original draft. CP, ND, JH, and DG reviewed and edited the original draft. All authors contributed to the article and approved the submitted version.

## Funding

This project was supported in part by the Initiative for the development of new technologies and innovative practices in rehabilitation (INSPIRE) and a Canadian Institutes of Health Research Grant to ND (CIHR #389886). CP holds a scholarship from INSPIRE and the School of Rehabilitation (*Bourse de Mérite de la Faculté de médecine de l'Université de Montréal*), ML holds a scholarship from the Université de Montréal (Bourse du PRogramme d'Excellence en Médecine pour l'Initiation En Recherche PREMIER de la Faculté de médecine de l'Université de Montréal) and DG and ND hold senior research salary award from the Fonds de la recherche du Québec – Santé (FRQS; #268982 and #252479, respectively).

## Conflict of Interest

The authors declare that the research was conducted in the absence of any commercial or financial relationships that could be construed as a potential conflict of interest.

## Publisher's Note

All claims expressed in this article are solely those of the authors and do not necessarily represent those of their affiliated organizations, or those of the publisher, the editors and the reviewers. Any product that may be evaluated in this article, or claim that may be made by its manufacturer, is not guaranteed or endorsed by the publisher.
